# Tunable Viscoelastic Properties of Sodium Polyacrylate Solution via CO_2_-Responsive Switchable Water

**DOI:** 10.3390/molecules26133840

**Published:** 2021-06-24

**Authors:** Dianguo Wu, Yiwen Shi, Kun Lv, Bing Wei, Youyi Zhu, Hongyao Yin, Yujun Feng

**Affiliations:** 1 State Key Laboratory of Oil and Gas Reservoir Geology and Exploitation, Southwest Petroleum University, Chengdu 610500, China; wu-dianguo@foxmail.com (D.W.); bwei@swpu.edu.cn (B.W.); 2 Polymer Research Institute, Sichuan University, Chengdu 610065, China; lyukun@stu.scu.edu.cn; 3 Pittsburgh Institute, Sichuan University, Chengdu 610065, China; sywvivi@hotmail.com; 4 Research Institute of Petroleum Exploration and Development, CNPC, Beijing 100083, China; zhyy@petrochina.com.cn

**Keywords:** viscoelastic fluids, CO_2_-switchable, sodium polyacrylate, *N,N*-dimethyl ethanol amine

## Abstract

Upon stimulus by CO_2_, CO_2_-switchable viscoelastic fluids experience a deliberate transition between non-viscous and highly viscous solution states. Despite attracting considerable recent attention, most such fluids have not been applied at a large- scale due to their high costs and/or complex synthesis processes. Here, we report the development of CO_2_-switchable viscoelastic fluids using commercially available sodium polyacrylate (NaPAA) and *N*,*N*-dimethyl ethanol amine (DMEA)-based switchable water. Upon bubbling CO_2_, into the solutions under study, DMEA molecules are protonated to generate quaternary ammonium salts, resulting in pronounced decreases in solutions viscosity and elasticity due to the influence of increased ionic strength on NaPAA molecular conformations. Upon removal of CO_2_ via introduction of N_2_, quaternary salts are deprotonated to tertiary amines, allowing recovery of fluid viscosity and elasticity to near the initial state. This work provides a simple approach to fabricating CO_2_-switchable viscoelastic fluids, widening the potential use of CO_2_ in stimuli-responsive applications.

## 1. Introduction

As a non-Newtonian fluid, a viscoelastic solution usually exhibits unique rheological properties (i.e., both liquid-like fluidity under some circumstances and solid-like elasticity under others) [1,2,3,4], which endow it with many distinct behaviors, such as the Weissenberg effect [2,5], extrudate swell [3], and fading memory [6]. Those characteristics allow for great potential applications in fields like food science [4], damping [7], tissue engineering [8], and oil development [9,10]. Over the past few decades, smart viscoelastic fluids that respond reversibly to environmental stimuli, e.g., temperature [11,12,13,14,15], pH [16,17], light [18], and CO_2_ [19], to demonstrate tunable rheological properties have drawn extensive attention both from engineers in industry and fundamental theoretical scientists. Among above triggers, CO_2_ has garnered considerable interests recently due to its nontoxicity, low cost, high availability, and good biocompatibility [19,20,21,22]. Generally, CO_2_-responsive viscoelastic systems can experience a purposeful alteration from non-viscous liquid to high-viscosity solution or gel, by the stimulus of CO_2_. As of yet, many different CO_2_-responsive viscoelastic fluids have been reported, often utilizing a reversible reaction between CO_2_ and guanidines, amidines, or amines [21].

So far, CO_2_-responsive viscoelastic systems based on surfactants and/or polymers have been developed. Surfactant wormlike micelles (WLMs) are long flexible aggregates that can entangle to form three-dimensional networks that impart remarkable viscoelastic properties to the bulk solution. Thus, the reversible generation and destruction of wormlike micelles via external stimulations can realize switchable viscoelastic fluids [23]. Feng and co-workers [23] pioneered CO_2_-switchable WLMs using sodium dodecyl sulfate (SDS) and *N*,*N*,*N*′,*N*′-tetramethyl-1,3-propanediamine (TMPDA). CO_2_ was shown to protonate tertiary amino groups of TMPDA, which then interacted with SDS to generate WLMs. Thus, the alternating introduction and removal of CO_2_ resulted in a reversible viscoelastic fluid.

In addition to surfactant, water-soluble polymers, including synthetic and natural polymers, is another class of important material preparing viscoelastic fluids, which are widely used as rheology modifiers in the oil and gas industry. The strategy to construct CO_2_-switchable polymer viscoelastic fluid is usually to copolymerize a water-soluble species of monomer with a CO_2_-sensitive monomer, such as *N*,*N*-dimethylaminoethyl methacrylate (DMAEMA) [19,24] and *N*,*N*-diethylaminoethyl methacrylate (DEAEMA) [25]. The protonation and de-protonation of the tertiary amino group in these monomers in the presence and absence, respectively, of CO_2_ lead to a large change in hydrophilicity, which gives rise to variations in the fluid’s viscoelasticity. However, a complicated synthesis process as well as high cost of the CO_2_-sensitive monomer make large-scale industrial applications difficult to achieve. Therefore, it is highly desirable to find an inexpensive and simple system for the fabrication of CO_2_-switchable viscoelastic fluids.

Sodium polyacrylate (NaPAA) is a typical polyelectrolyte, possessing many charged carboxylate groups along the main polymer chain upon its dissociation in water. Due to these groups, electrostatic repulsions arise between inter- and intramolecular chains, imparting high viscosity and elasticity to the aqueous solution. This strong thickening power as well as its low cost and easy availability facilitates wide use of NaPAA in industry. However, electrostatic repulsions between the charged groups of NaPAA are susceptible to interference from salts. Fujita et al. [26] revealed that an apparent decrease occurred in the viscosity of NaPAA solutions when sodium chloride (NaCl) was added, because the corresponding increase in ionic strength of the solution allowed PAA molecules to coil up more and more tightly. Klaus [27] found that the addition of Ca^2+^ to NaPAA solution similarly caused polymer coils to apparently shrink, resulting in dramatic viscosity decreases with incremental introduction of Ca^2+^. It has thus become clear that the rheological properties of NaPAA aqueous solution are highly sensitive to ionic strength. Therefore, a solution that incorporates switchable ionic strength is expected to impart the NaPAA fluid with smart, tunable rheological properties. Jessop and co-workers [28,29,30] pioneered the development of an aqueous solution of various amines, called “switchable water”, which demonstrated CO_2_-switchable ionic strength. In the absence of CO_2_, the ionic strength of switchable water was very low, but in the presence of CO_2_, the amines were converted into the bicarbonate salts, resulting in a dramatic and reversible rise in ionic strength.

The aim of this work is to reversibly regulate the rheological properties of NaPAA-based solution via the incorporation of switchable water. Herein, *N,N*-dimethyl ethanol amine (DMEA) was chosen as an additive to prepare CO_2_-switchable water, and the rheological properties of NaPAA in the switchable water with and without CO_2_ were investigated. Both viscosity and elasticity of the solutions were found to decrease after CO_2_-treatment, and then were partially recovered once CO_2_ was removed by bubbling N_2_ at elevated temperature. The effects of NaPAA and DMEA concentrations on the variation of rheological properties were examined and the corresponding mechanism behind was also discussed.

## 2. Results and Discussion

### 2.1. Rheological Properties of NaPAA Aqueous Solutions and Their CO_2_-Responsive Behavior

**Figure 1:**
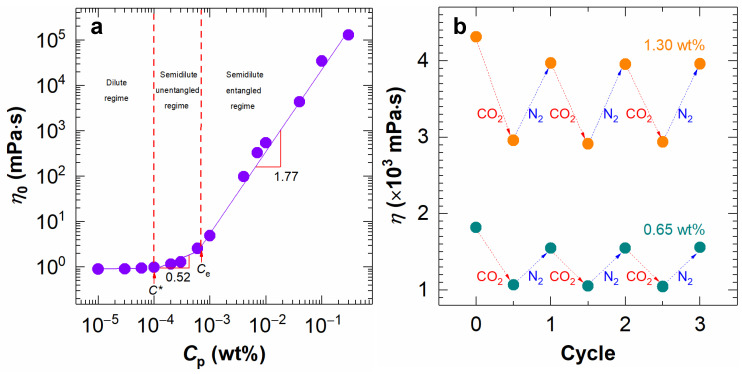
(**a**) Variation of *η*_0_ with NaPAA concentration in pure water, and (**b**) the viscosity alternation of 0.65 and 1.30 wt% NaPAA aqueous solutions at shear rate of 10 s^−1^ upon alternately bubbling CO_2_ and N_2_ at 25 °C.

Owing to its strong thickening power and easy availability, NaPAA is widely used in various industries as rheology modifier. The NaPAA used in this work was a commercial product with *M*_W_ of 4 × 10^6^–5 × 10^6^ g·mol^−1^, and its thickening ability was investigated. A series of aqueous solutions with different NaPAA concentrations were prepared, and the viscosity-shear rate curves (Figure S1) of each were measured to obtain the zero-shear viscosity (*η*_0_), which is the value when the shear rate tends to zero. Figure 1a shows a plot of *η*_0_ at varying concentrations of NaPAA in pure water. One can find that the curve has been divided into three concentration regimes with two clear breakpoints. At low concentrations, the NaPAA aqueous solutions exhibited Newtonian fluids properties, and the *η*_0_ was found to be very close to that of the solvent, classifying these fluids as dilute [31]. The critical overlap concentrations (C*), at which the solution transitions from dilute to semidilute behavior, were determined from the concentrations at which the measured solution viscosity began to abruptly increase. Herein, C* (≈ 0.0001 wt%) falls in the second area shown in Figure 1a, in which the solution viscosity was found to increase with the increment of NaPAA concentration according to a power law with an exponent of 0.52, approaching a theoretically predicted value (0.5) for semidilute, unentangled polyelectrolyte solutions in previous studies [32,33,34]. In the third region of Figure 1a, *η*_0_ of the fluids increased more dramatically with concentration than it did in semidilute, unentangled regime, demonstrating a power law with an exponent of 1.77, near the expected power index (1.5) of semidilute, entangled polyelectrolyte fluids [33,34]. Based on the semidilute unentangled and entangled regime scaling, the second turning point was defined as the critical entanglement concentration, *i.e.*, *C*_e_ (≈ 0.0007 wt%), as labeled in Figure 1a.

To investigate the influence of CO_2_ on the NaPAA fluid, two different concentrations of NaPAA, 0.65 and 1.30 wt%, representing solutions with high viscosity and elasticity in the semidilute entangled regime, were selected. The NaPAA aqueous solutions with concentrations of 0.65 wt% and 1.30 wt% were prepared, and their rheological behaviors were examined at 25 °C under steady and dynamic shear conditions. Figure S2 shows the viscosity-shear rate curves of the samples containing 0.65 wt% and 1.30 wt% NaPAA in the absence and presence of CO_2_. It can be found that the fluids showed Newtonian behavior with high, near-constant viscosities at low shear rates, then exhibited shear thinning properties in which the viscosities sharply decreased with the increment in shear rate, which was consistent with typical rheological properties of viscoelastic fluids [3,9]. After bubbling CO_2_ for 10 min until the conductivity of the solutions stabilized, the viscosity of each fluid underwent an apparent decrease over the entire range of tested shear rates, and the lower the shear rate, the larger the decrement in viscosity.

N_2_ was subsequently introduced into the solutions at 60 °C to remove CO_2_, and the viscosities of both the 0.65 wt% and 1.30 wt% NaPAA solution were partially recovered. Figure 1b presents the variation in viscosity that arose for each fluid at a shear rate of 10 s^−1^ upon alternately bubbling CO_2_ and N_2_; both solutions exhibited a clear CO_2_-responsive change in viscosity. For the 0.65 wt% NaPAA solution, its viscosity was initially 1821 mPa·s, and dropped sharply to 1056 mPa·s upon CO_2_ treatment, then increased to 1553 mPa·s when N_2_ was bubbled in. In contrast, the initial viscosity of the 1.30 wt% NaPAA in pure water decreased from 4314 mPa·s to 2956 mPa·s after treating with CO_2_, then recovered to 3962 mPa·s when bubbling N_2_. For the 0.65 wt% and 1.30 wt% NaPAA aqueous solutions at a shear rate of 10 s^−1^, the degrees of viscosity recovery after alternating CO_2_/N_2_ treatments were 85% and 92%, respectively. These results clearly indicate that alternating addition and removal of CO_2_ facilitated a reversible change in the viscosities of the NaPAA aqueous solutions.

**Figure 2:**
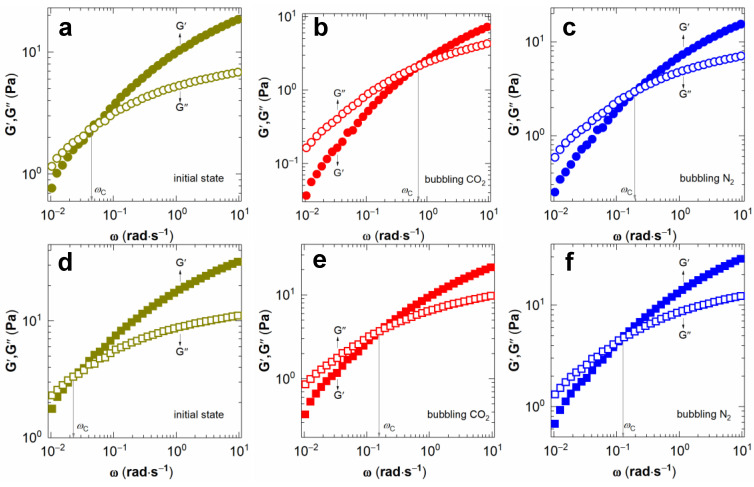
Storage modulus G′ and loss modulus G″ as a function of frequency for (**a**) 0.65 wt% and (**d**) 1.30 wt% NaPAA aqueous solutions in initial state, (**b**) 0.65 wt% and (**e**) 1.30 wt% NaPAA aqueous solutions after bubbling CO_2_, and (**c**) 0.65 wt% and (**f**) 1.30 wt% NaPAA aqueous solutions when treated by N_2_ to remove CO_2_. All tests were conducted at a shear strain of 10% at 25 °C.

The effect of CO_2_ on the viscoelasticity of the NaPAA aqueous solutions was then investigated via oscillatory-shear measurements. Before measurements, the linear viscoelastic region of the NaPAA aqueous solutions was confirmed through a strain sweep test. As shown in Figure S3, at strains lower than 40 % at 25 °C, the fluids exhibited linear viscoelastic behavior, defining the region in which the solutions could retain a stable structure without being destroyed [35]. Here, storage modulus (G′) characterized elastic behavior of the NaPAA solutions, while loss modulus (G″) represented its viscous properties. Under low strain, G′ > G″, for both samples, indicating that they exhibited solid gel-like behaviors. Contrarily, in the high strain regime, G′ < G″, demonstrating that the both samples showed fluid-like properties. Therefore, frequency sweep curves for the 0.65 wt% and 1.30 wt% NaPAA aqueous solutions were obtained at a strain of 10 %, as shown in Figure 2.

Regardless of whether CO_2_ was present, the solutions displayed classic viscoelastic character, i.e., they showed a crossover of G′ and G″ within the tested range of strain frequencies. The maximum relaxation time (τ_R_) for a viscoelastic fluid is generally defined as the inverse of this intersectant frequency (*ω*_c_) [36]; thus, the fluid’s response can be divided into two regimes based on *τ*_R_. At low frequency (*ω* << *ω*_c_), G′ < G″, indicating that samples possessed a viscous behavior; while at high frequency (*ω* >> *ω*_c_), G′ > G″, the fluids behaved an elastic property. Additionally, for the 0.65 wt% NaPAA aqueous solution, the point of intersection shifted to a higher frequency after CO_2_ treatment, corresponding to a decrease in *τ*_R_ from ~25 s to ~1.45 s, then recovered to a lower frequency upon bubbling N_2_, i.e., *τ*_R_ increased to ~5.26 s. In comparison, *τ*_R_ of the 1.30 wt% NaPAA aqueous solution decreased from ~50 s to ~5.56 s after bubbling CO_2_, and then increased to ~8.33 s with the introduction of N_2_. These results clearly suggest that the viscoelastic character of NaPAA aqueous solution can be reversibly tuned through alternating introduction of CO_2_ and N_2_. Moreover, both G′ and G″ were dependent on frequency and much lower in the presence of CO_2_, confirming that the introduction of CO_2_ weakened the strength of the fluids.

### 2.2. Effect of DMEA CO_2_-Switchable Water on Rheological Properties of NaPAA Solutions

DMEA contains a tertiary amino group that can be well dissolved in water and protonated by CO_2_ to produce ammonium bicarbonate salt, yielding a large increase in ionic strength of the aqueous solution. The protonated product can be converted back into to the original amine by bubbling N_2_, showing good reversibility [22,37]. For these reasons, DMEA was chosen to prepare CO_2_-switchable water, and the effect of both DEMA concentration and the introduction of CO_2_ on the rheological behavior of NaPAA aqueous solution was investigated.

**Figure 3:**
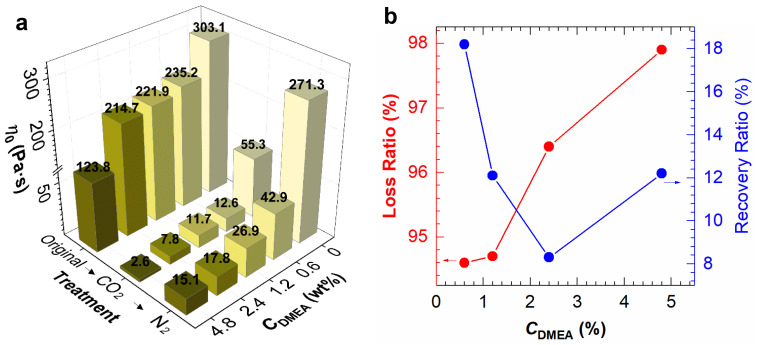
(**a**) Variation in *η*_0_ of 0.65 wt% NaPAA solution with different DMEA concentrations before and after bubbling CO_2_ and N_2_, and (**b**) loss and recovery ratios of *η*_0_ for 0.65 wt% NaPAA solution with different DMEA concentration after treatment of CO_2_ and N_2_ at 25 °C.

The influence of different DMEA concentrations on the steady rheology properties of 0.65 wt% and 1.30 wt% NaPAA solutions in the presence and absence of CO_2_ is shown in Figures S4 and S5, respectively. One can find that the initial solution showed the largest viscosities in low shear rate region, while the CO_2_-treated solutions exhibited the lowest viscosities at the same shear rate. Values of *η*_0_ were obtained from Figures S4 and S5, and further plotted against DMEA concentrations. As displayed in Figure 3a, *η*_0_ of the 0.65% NaPAA aqueous solution without the addition of DMEA was initially 303.1 Pa·s^−1^, then decreased to 55.3 Pa·s^−1^ in the presence of CO_2_, and recovered to 273.1 Pa·s^−1^ after introducing N_2_.

Upon adding DMEA to the 0.65% NaPAA aqueous solution, the initial *η*_0_ of the 0.65wt% NaPAA solution decreased with the increment of DMEA, which can be interpreted as the introduction of DMEA increased the ionic strength of the mixed solution, weakening electrostatic repulsions between NaPAA chains. While bubbling CO_2_, tertiary amino groups in DMEA reacted with CO_2_ to produce ammonium bicarbonate salts, which further remarkably increased the ionic strength of the solution, resulting in a sharp decrease in *η*_0_ of the 0.65 wt% NaPAA solutions. With respect to the CO_2_-treated solutions, varying DMEA concentration from 0.6 wt% to 4.8 wt% led to decreases in *η*_0_ from 12.6 Pa·s^−1^ to 2.6 Pa·s^−1^. Bubbling N_2_ to remove CO_2_ led to partial recovery of the initial solution viscosity across all concentrations of DMEA, as shown in Figure 3a.

To investigate the effect of CO_2_ on *η*_0_ in the presence of DMEA, a loss ratio index, i.e., the ratio of lost *η*_0_ in CO_2_-treated solution to its initial value, was introduced, as displayed in Figure 3b. The data indicates that the loss ratio of *η*_0_ upon introduction of CO_2_ increased from 94.6 % to 97.9 % with the increment of DMEA, implying that the protonated DMEA could greatly weaken the viscosity of NaPAA solution, demonstrating behavior similar to that of Na^+^ and Ca^2+^ in prior studies [26,27]. However, unlike Na^+^ and Ca^2+^, the loss of NaPAA solution viscosity induced by protonated DMEA could be reversed to some extent, as shown in Figure 3a. Therefore, to assess the degree of *η*_0_ that occurred after introducing N_2_, a recovery ratio, i.e., the ratio of *η*_0_ of the N_2_-treated solution to its initial value, is also presented in Figure 3b. It can be found that the recovery ratio varied between 8.3 % and 18.2 %, indicating that the DMEA was not completely deprotonated by bubbling N_2_ at 60 °C.

The influence of different DMEA concentrations on *η*_0_ of the 1.30 wt% NaPAA solution, in the presence or absence of CO_2_, is displayed in Figure S6. The variation of *η*_0_ with concentrations of DMEA exhibited similar tendencies to that of the 0.65 wt% NaPAA solution. However, the loss ratio in *η*_0_ of 1.30 wt% NaPAA solution was lower than that of 0.65 wt% solution, and the recovery ratio in *η*_0_ of 1.30 wt% NaPAA solution was higher than that of 0.65 wt% solution, indicating that the inter- and intramolecular entanglement of 1.30 wt% NaPAA solution is stronger than that of 0.65 wt% solution. These results prove that the viscosity of NaPAA solutions could be reversibly tuned via the protonation and deprotonation of DMEA.

**Figure 4:**
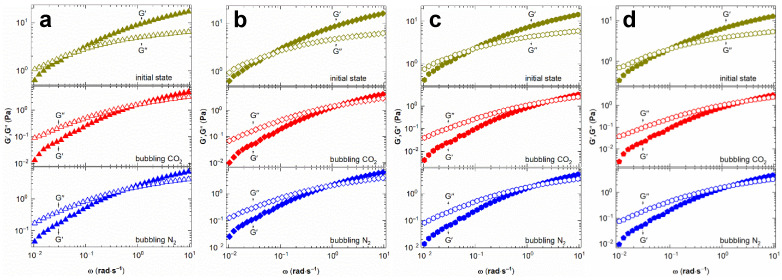
Frequency sweep curves of 0.65 wt% NaPAA aqueous solutions containing (**a**) 0.60 wt%, (**b**) 1.20 wt%, (**c**) 2.40 wt%, and (**d**) 4.80 wt% DMEA before and after treatment of CO_2_ and N_2_, measured at shear strain of 10 % at 25 °C.

**Figure 5:**
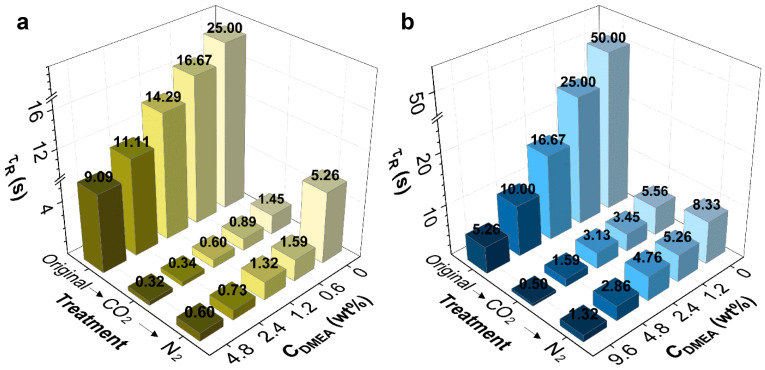
Variation in maximum relaxation time *τ*_R_ of (**a**) 0.65 wt%, and (**b**) 1.30 wt% NaPAA solutions with different DMEA concentrations before and after bubbling CO_2_ and N_2_ at 25 °C.

Then, the influence of DMEA concentration on the dynamic rheological behavior of NaPAA fluids upon CO_2_/N_2_ treatment was also investigated. Figure 4 presents frequency sweep curves of 0.65 wt% NaPAA solutions with different DMEA contents. As mentioned previously, the G′/G″-crossover point on the frequency sweep curves provides the frequency of the viscous-to-elastic transition corresponding to the maximum relaxation time, *τ*_R_. Measured values of *τ*_R_ are plotted against variations in DMEA concentration upon alternate gas treatments in Figure 5.

Figure 5a demonstrates that, for the 0.65 wt% NaPAA solutions, the initial *τ*_R_ gradually decreased from 25.00 s to 9.09 s with the DMEA content increased from 0 to 4.80 wt%. Thus, the strength of the NaPAA fluids was weakened by the addition of DMEA. Upon treatment of CO_2_ until conductivity stabilized, DMEA was protonated by CO_2_, resulting in the presence of quaternary ammonium salts in the solution [22,37]. Consequently, *τ*_R_ of the solutions further declined from 1.45 s to 0.32 s with increasing DMEA concentrations, suggesting that protonated DMEA enhanced CO_2_-induced weaking of the fluid. To remove CO_2_, N_2_ was introduced into the mixture, and *τ*_R_ was partially recovered to its initial value, again proving that the quaternary ammonium salts of DMEA were only partially deprotonated by N_2_ treatment.

The variation of *τ*_R_ with different DMEA concentrations obtained for the 1.30 wt% NAPAA solution exhibited a similar variation tendency upon alternately bubbling CO_2_ and N_2_, as shown in Figure S7 and Figure 5b. These results illustrate that the viscoelasticity of NaPAA solutions could be reversibly adjusted through the incorporation of DMEA-based CO_2_-switchable water.

### 2.3. Mechanism of CO_2_-Switchable Viscoelasticity

Rheological characterizations showed that CO_2_ treatment could decrease the viscosity and weaken the elasticity of NaPAA fluids, which might be ascribe to the reaction between incorporated DMEA and CO_2_. To elucidate the corresponding mechanism, the pH and conductivity of NaPAA solutions with different DMEA concentrations were continuously monitored during cyclical CO_2_/N_2_ treatment at 25 °C.

**Scheme 1:**
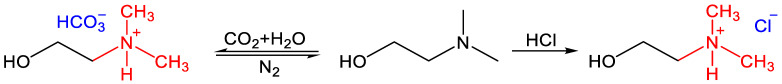
Protonation of DMEA with HCl or CO_2_.

**Figure 6:**
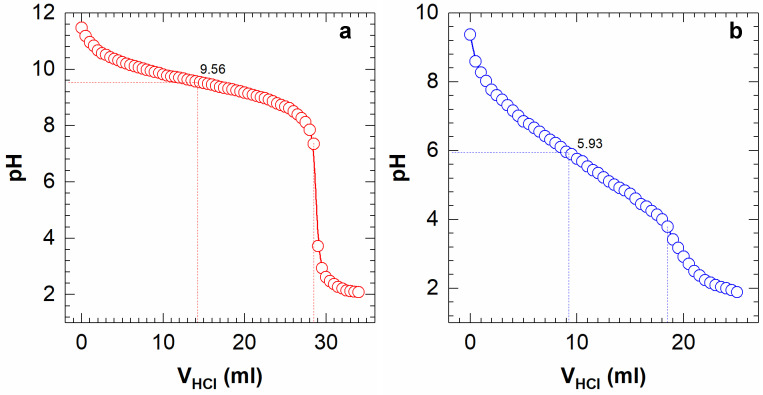
pH titration curves of (**a**) DMEA and (**b**) NaPAA aqueous solutions against 0.1 M HCl at 25 °C.

In general, the basicity of functional groups can be judged based on the dissociation constant (p*K*_aH_) of its conjugate acid; the larger the p*K*_aH_, the stronger the basicity [20]. The pH titration curves of aqueous solutions of DMEA and NaPAA against HCl solution were obtained to determine the p*K*_aH_ of DMEA and NaPAA. In a typical titration curve, the pH corresponding to the half equivalence points is taken as the average p*K*_aH_ [20]. The reaction between DMEA and HCl is exhibited in Scheme 1, and the titration curve of the DMEA solution is shown in Figure 6a, in which the p*K*_aH_ of DMEA was found to be 9.56. Similarly, the p*K*_aH_ of NaPAA used in this study was found to be 5.93, as shown in Figure 6b.

In terms of the p*K*_aH_ values determined above, the degree of protonation (*δ*), i.e., the proportion of protonated organ-base, can be calculated using Equations (1) and (2) [20,38].

(1) $$Ka=\frac{{[M][H}^{+}]}{{[MH}^{+}]}$$

(2) $$\delta =\frac{1}{{1+10}^{pH-pKa}}\;\times \;100\%$$

*[M]* in Equation (1) represents the concentration of DMEA or NaPAA, while *[MH^+^]* denotes the concentration of protonated DMEA or NaPAA.

**Figure 7:**
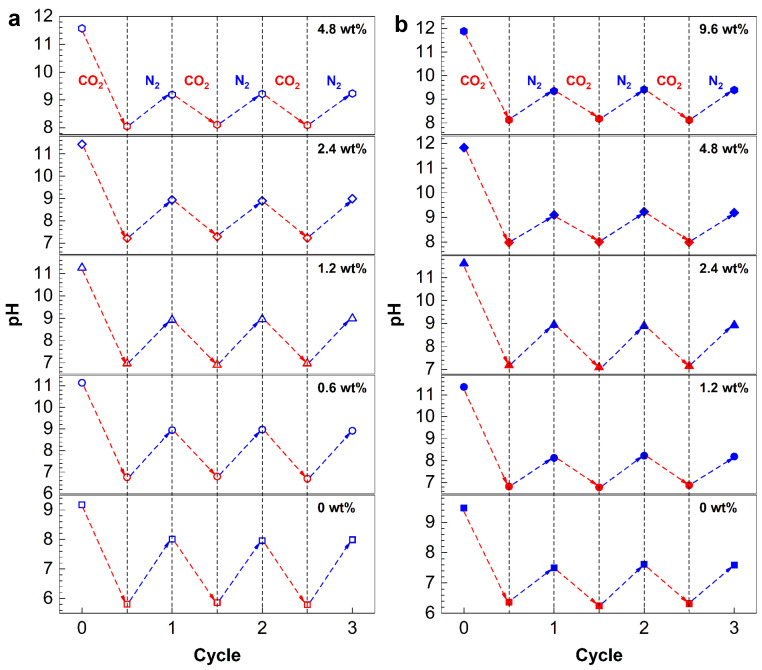
Variation of pH for (**a**) 0.65 wt% and (**b**) 1.30 wt% NaPAA solution at different DMEA concentration while alternatively bubbling CO_2_ and N_2_ at 25 °C.

Upon bubbling CO_2_, the pH of the 0.65 wt% NaPAA aqueous solution decreased from 9.17 to 5.82, as displayed in Figure 7a. According to Equation (2), the initial *δ* of NaPAA was ~0.06 mol%. The change in pH that occurred after sparging with CO_2_ indicates that *δ* increased to ~56.30 mol%, and thus ~56.24 mol% carboxylate groups were more protonated in the presence of CO_2_. These results coincide with results of the rheology experiments: viscosities *η* and relaxation times *τ*_R_ of the 0.65 wt% aqueous solutions dropped sharply after bubbling CO_2_, revealing that protonation of carboxylate side groups reduced the charge density and corresponding electrostatic repulsions between NaPAA chains to allow a smaller average molecular size than in the initial state. After bubbling N_2_, the pH of 0.65 wt% NaPAA aqueous solution recovered to 7.99, signifying deprotonation of the carboxylate groups. The pH was not totally restored to its initial value, indicating that 0.86 mol% carboxylate groups were still protonated. This phenomenon also coincides with the prior rheological results, in which the viscosities *η* and relaxation times *τ*_R_ of NaPAA aqueous solutions were not fully restored to their initial values.

The pH of 0.65 wt% NaPAA solutions containing different DMEA concentrations was also examined (Figure 7a). It can be found that the pH of 0.65 wt% NaPAA solutions increased apparently with the increment of *C*_DMEA_, regardless of the presence of CO_2_ (Table S1). As an example, the variation in pH for the 0.65 wt% NaPAA solution with 0.6 wt% DMEA upon the introduction of CO_2_ and N_2_ is discussed. As a typical tertiary amine, DMEA can be protonated in water in the presence of CO_2_ to generate ammonium bicarbonate salts (Scheme 1) [39,40,41,42]. The initial pH of 11.14 was reduced to 6.75 upon bubbling CO_2_, and then recovered to 8.94 after introducing N_2_. Via Equation (2), the initial *δ* of DMEA can be calculated at ~2.56 mol%, followed by an increase to ~99.85 mol% after the introduction of CO_2_, indicating that the tertiary amino groups on DMEA molecules were almost completely protonated. After bubbling N_2_, the pH of the solution did not recover to its initial value; rather, 80.65 mol% of the tertiary groups were still protonated, which was caused by the larger the basicity, the poorer the “switch” capability [20]. Besides, the initial *δ* for NaPAA of 6.17 × 10^−4^ mol% was increased to 13.15 mol% after bubbling CO_2_, then again restored to 9.76 × 10^−2^ mol%. Thus, one can find that even if CO_2_ is not available, there was at least some small amount of protonated DMEA in the solution. These results properly clarify why viscoelasticity of the NaPAA solutions containing DMEA evidently decreased before bubbling CO_2_. By contrast, in the presence of CO_2_, a large number of protonated DMEA increased the charge density of the fluids, together with protonated carboxylate groups reduced the charge density and electrostatic repulsions between molecular chains, then the synergy of the two effects diminished the size of NaPAA molecules, resulting in the decrease of *η* and *τ*_R_ with the addition of DMEA. The change in pH of 1.30 wt% NaPAA solution with different DMEA concentrations demonstrated similar tendencies, as shown in Figure 7b and Table S2.

**Figure 8:**
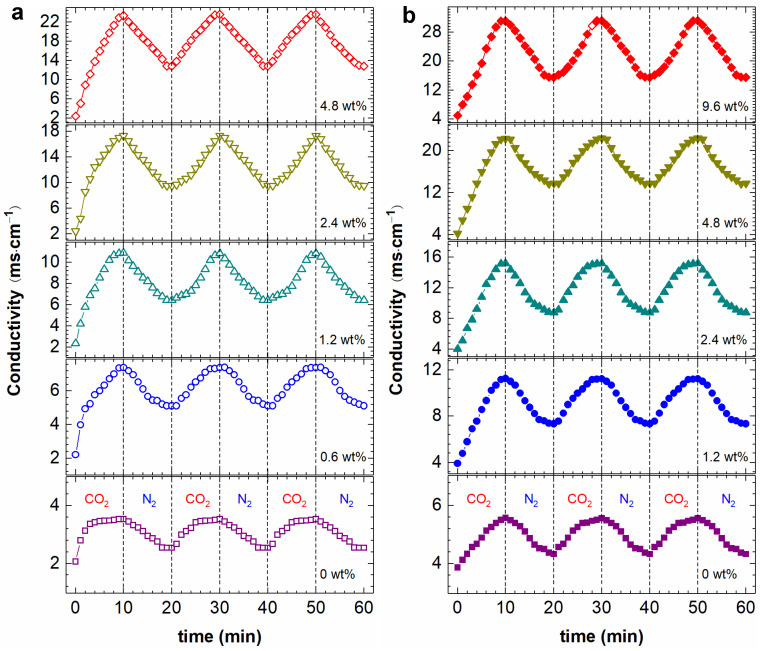
Variation in conductivity of (**a**) 0.65 wt% and (**b**) 1.30 wt% NaPAA solutions at different DMEA concentrations, while alternatively bubbling CO_2_ and N_2_ at 25 °C.

As a typical polyelectrolyte, the conformation of NaPAA molecule chains is usually affected by salts in solutions, which influences the rheological properties of the fluids. To verify the changes in charge density in the NaPAA/DMEA solution at different states, Figure 8a provides conductivity of 0.65 wt% solution with different DMEA concentrations in the presence or absence of CO_2_ at 25 °C. The conductivity of the 0.65 wt% NaPAA aqueous solution was initially 2.06 mS·cm^−1^, then increased to 3.53 mS·cm^−1^ upon CO_2_ treatment, suggesting that various inorganic ions (H^+^, HCO_3_^−^, CO_3_^2−^) were produced. After bubbling N_2_ to remove CO_2_, the conductivity of the 0.65 wt% aqueous solution returned to 2.54 mS·cm^−1^, which was very close to its initial value. Hence, the enhancement of ionic strength weakened electrostatic repulsions between inter- and intramolecular chains, resulting in the observed drop in *η* and *τ*_R_.

Besides, the protonated DMEA, i.e., DMEAH^+^, further increased the ionic strength of the fluids. Data shown in Figure 8a demonstrates that, when bubbling CO_2_, the maximum conductivity of 0.65 wt% NaPAA solution increased with the addition of DMEA, implying that DMEAH^+^ was generated in those fluids. The charged DMEAH^+^ molecules shielded static repulsions among carboxylate groups (–COO^−^) of the NaPAA molecules, allowing the polymer chains to become coiled and thus reducing electrostatic repulsions between them. Thus, in the presence of CO_2_, the *η* and *τ*_R_ of 0.65 wt% NaPAA solution decreased with the increment of DMEA. After introducing N_2_, the conductivity of the fluids partially returned to their initial values, as exhibited in Figure 8a and Table S3. These results properly explain why *η* and *τ*_R_ could not completely go back to their initial values when introducing N_2_ to remove CO_2_. Three repeated cycles indicated that DMEA possesses good CO_2_-induced switching behavior. In addition, the data in Figure 8b and Table S4 also show that variation in conductivity of the 1.30 wt% NaPAA soution with different DMEA concentrations also obeyed the same rule.

**Scheme 2:**
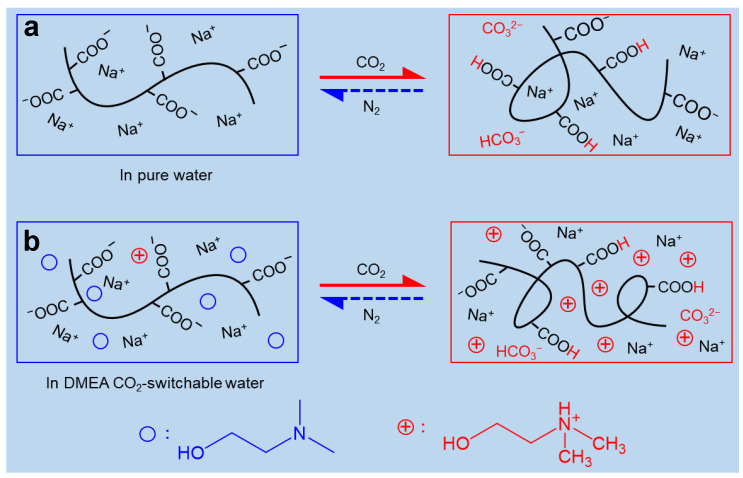
Schematic illustration of the mechanism for CO_2_-switchable viscoelasticity of NaPAA in (**a**) pure water, and (**b**) DMEA solution.

Consequently, the mechanism for CO_2_-switchable rheological behavior of NaPAA solutions, with or without the addition of DMEA, can be summarized in Scheme 2. On the one hand, for NaPAA aqueous solution, bubbling CO_2_ leads to –COO^−^ in side chains will be protonated to carboxylic acid (–COOH), resulting in a decrease in charge density on the molecular chains relative to that of the initial solution state, as shown in Scheme 2a. Therefore, electrostatic repulsions between intra- and inter-molecules due to –COO^−^ groups are weakened, and the viscoelasticity of the NaPAA aqueous solution decreases accordingly. Treatment with N_2_ allows viscoelasticity of the solution to partially recover, due to the partial deprotonation of –COOH groups. On the other hand, for NaPAA solutions that contain DMEA, in addition to the influence of protonation of –COO^−^, the protonated DMEA plays a crucial role in determining the viscoelasticity of the solution upon the addition of CO_2_, as displayed in Scheme 2b. When CO_2_ is bubbled into the solutions, the DMEA molecules are protonated to generate quaternary ammonium salts, which shield charges remaining on the NaPAA chains, allowing them to coil more tightly, resulting in a sharp decrease in viscoelasticity of the fluids. Upon removal of CO_2_, the quaternary salts are deprotonated, causing partial recovery of the viscosity and elasticity of the solutions.

## 3. Materials and Methods

### 3.1. Materials

NaPAA (*M*_W_ = 4 × 10^6^–5 × 10^6^ g·mol^−1^) and DMEA (≥99%) were purchased from Sigma-Aldrich and used as received. CO_2_ (≥99.998%) and N_2_ (≥99.998%) were supplied by Xuyuan Chemical Industry Co., Ltd. (Chengdu, China) and used without further treatment. Ultrapure water with resistivity of 18.25 MΩ cm^−1^ was produced by an ultrapure water purification system (Chengdu Ultrapure Technology Co., Ltd., Chengdu, China) and used throughout this study.

### 3.2. Sample Preparation

A general method of preparing NaPAA-DMEA aqueous solutions is described as follows. First, a designated amount of NaPAA was added to a beaker with ultrapure water, and the mixture was stirred until NaPAA was completely dissolved. Second, a designated amount of DMEA was completely dissolved in ultrapure water. Then, the two solutions were mixed and stirred for 24 h at room temperature to produce the final solution.

#### 3.2.1. CO_2_ Treatment

CO_2_ gas was bubbled into the fluid via a syringe needle at 25 °C, until the measured conductivity of the solution stabilized.

#### 3.2.2. N_2_ Treatment

To remove CO_2_, N_2_ gas was bubbled into the previously CO_2_-treated fluid via a syringe needle at 60 °C, until the measured conductivity of the solution remains stable.

### 3.3. Rheological Tests

Both steady and dynamic rheological measurements were performed via a Physica MCR 302 rotational rheometer (Anton Paar, Rannachstrasse, Austria) equipped with concentric cylinder geometry (CC27). All measurements were carried out in stress-controlled mode at 25 °C, and Cannon standard oil was used to calibrate the instrument before experimentation. All samples were centrifuged to eliminate the interior bubbles prior to measurements, then equilibrated at 25 °C for at least 10 min prior to experimentation. Dynamic frequency spectra were conducted in the linear viscoelastic region, as determined from dynamic stress sweep measurements.

### 3.4. pK_aH_ Determination

The p*K*_aH_ (p*K*_a_ of the protonated species) values of DMEA and NaPAA were determined by titrating 20 mL of 0.1 M aqueous solutions with 0.1 M hydrochloric acid. The pH was continuously monitored at 25 °C with a S2-T Kit pH-meter (Mettler Toledo, ±0.01, Zurich, Switzerland) calibrated with standard buffer solution. The p*K*_aH_ values were obtained by taking the pH readings at the mid-point between two pH jumps.

### 3.5. Conductivity Measurements

The conductivity of NaPAA-DMEA solution was recorded with a S230-K conductometer (Mettler Toledo, Zurich, Switzerland) at 25 °C while bubbling CO_2_ or N_2_ alternatively.

## 4. Conclusions

In summary, the rheological properties of NaPAA aqueous solutions with different DMEA concentrations were investigated in the presence and absence of CO_2_. The effects of NaPAA and DMEA concentrations on measured solution properties were examined to elucidate the mechanism of CO_2_-responsive tunable rheological properties. It was found that NaPAA showed strong thickening power in pure water. The introduction/removal of CO_2_ imparted tunable viscoelasticity to the bulk solution, attributed to protonation/deprotonation of carboxylate groups and corresponding reduction/increase in charge density among molecular chains, allowing for reduction/increase in the size of solvated molecular chains. Moreover, the addition of DMEA gave rise to further reductions in viscoelasticity upon bubbling CO_2_, but initial values could be restored to some extent via N_2_ treatment. Besides, higher concentrations of DMEA corresponded to greater losses in viscoelasticity. The CO_2_-responsive switching behavior of DMEA-containing solutions was attributed to protonation of DMEA, which enabled NaPAA molecules to coil up more tightly, thus lowering the viscoelasticity, and to deprotonation of DMEAH^+^ via bubbling N_2_, which enabled partial recuperation of the viscoelasticity. This work not only widens the utilization of CO_2_ in preparing smart viscoelastic fluids but also demonstrates fabrication of such systems using a low-cost, industrial polymer and stimuli-responsive additives.

## Data Availability

The data presented in this study are available on request from the corresponding author.

## Supplementary Materials

The following are available online, Figure S1: viscosity as a function of shear rate for NaPAA aqueous solutions at 25 °C; Figure S2: viscosity-shear rate curves of 0.65 wt% and 1.30 wt% NaPAA solution while alternatively bubbling CO_2_ and N_2_ at 25 °C; Figure S3: strain sweep curves of 0.65 wt% and 1.30 wt% NaPAA aqueous solution at 25 °C; Figure S4: viscosity-shear rate curves of 0.65 wt% NaPAA solution with different DMEA concentrations while alternatively bubbling CO_2_ and N_2_ at 25 °C; Figure S5: viscosity-shear rate curves of 1.30 wt% NaPAA solution with different DMEA concentrations while alternatively bubbling CO_2_ and N_2_ at 25 °C; Figure S6: *η*_0_ of 1.30 wt% NaPAA solution with different DMEA concentrations before and after bubbling CO_2_ or N_2_, and the loss and recovery ratio for 1.30 wt% NaPAA solution with different DMEA concentrations at 25 °C after alternative treatment of CO_2_ and N_2_; Figure S7: frequency sweep curves of 1.30 wt% NaPAA solution with different DMEA concentrations while alternatively bubbling CO_2_ and N_2_ at 25 °C; Table S1: pH of 0.65 wt% NaPAA solutions with different DMEA concentration at different conditions; Table S2: pH of 1.30 wt% NaPAA solutions with different DMEA concentration at different conditions; Table S3: conductivity of 0.65 wt% NaPAA solutions with different DMEA concentration at different conditions; Table S4: conductivity of 1.30 wt% NaPAA solutions with different DMEA concentration at different conditions.

## Sample Availability

Samples of the compounds, including NaPAA and DMEA, are available from the authors.
