# Supplementary material for: Tunable Viscoelastic Properties of Sodium Polyacrylate Solution via CO2-Responsive Switchable Water
Source: Molecules. 2021 Jun 24;26(13):3840. doi: 10.3390/molecules26133840 (PMC8270326; doi:10.3390/molecules26133840)
Supplement: Supplementary file 1 [file molecules-26-03840-s001.zip › molecules-1260550-supplementary.pdf]

molecules

MDPI

Supporting Information *for*

Tunable Viscoelastic Properties of Sodium Polyacrylate Solution via CO_2-Responsive Switchable Water

Dianguo Wu [1,2], Yiwen Shi [3], Kun Lv [2], Bing Wei [1], Youyi Zhu [4], Hongyao Yin [2,*], Yujun Feng [2,*]

1 State Key Laboratory of Oil and Gas Reservoir Geology and Exploitation, Southwest Petroleum University, Chengdu 610500, People's Republic of China; wudianguo@foxmail.com (D.W.), bwei@swpu.edu.cn (B.W.)
2 Polymer Research Institute, Sichuan University, Chengdu 610065, People's Republic of China; lyukun@stu.scu.edu.cn (K.L.)
3 Sichuan University-Pittsburgh Institute, Sichuan University, Chengdu 610065, People's Republic of China; sywvivi@hotmail.com (Y.S.)
4 Research Institute of Petroleum Exploration and Development, CNPC, Beijing 10083, People's Republic of China; zhyy@petrochina.com.cn (Y.Z.)
* Correspondence: hyyin@scu.edu.cn (H. Yin); yjfeng@scu.edu.cn (Y. Feng)

S1. Viscosity-shear rate curves of NaPAA aqueous solution

The flow curves of NaPAA solution with different concentrations in deionized water are displayed in Figure S1. The shear viscosity increases with the increment of NaPAA concentrations. In addition, the NaPAA aqueous solutions show a Newtonian region initially, followed by a shear thinning behavior, which can be attributed to the increase in the alignment of the polymer molecules in the flow direction by shearing [1,2].

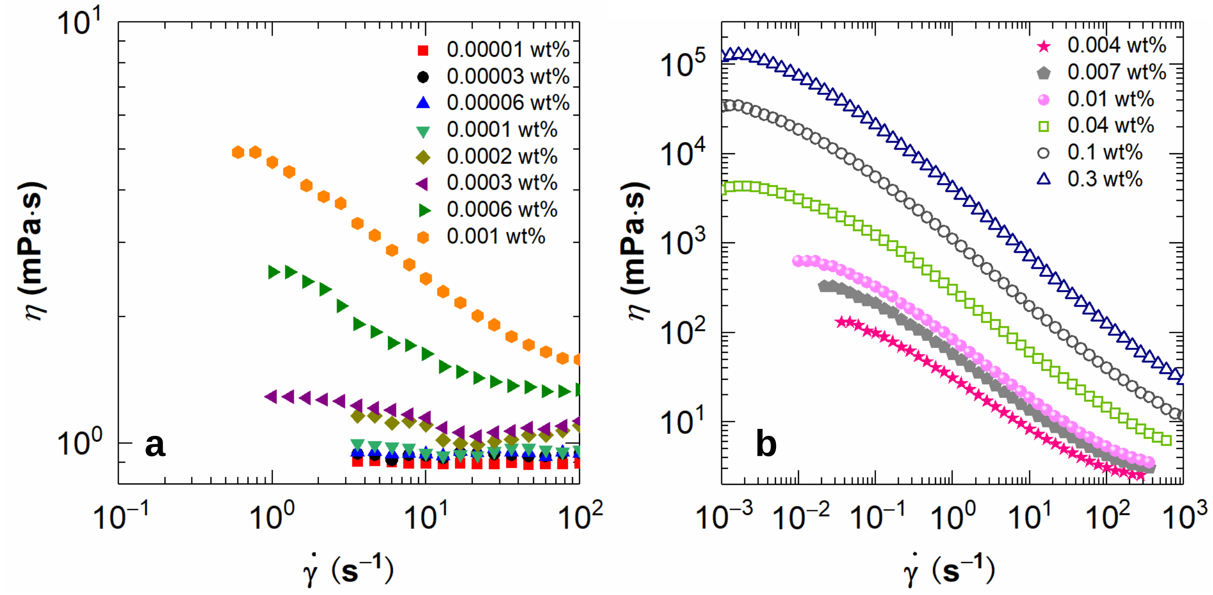

Figure S1 Viscosity as a function of shear rate for NaPAA aqueous solution at (a) low concentrations and (b) high concentrations at 25 °C.

S2: Viscosity-shear rate curves of 0.65 wt% and 1.30 wt% NaPAA aqueous solution at different conditions

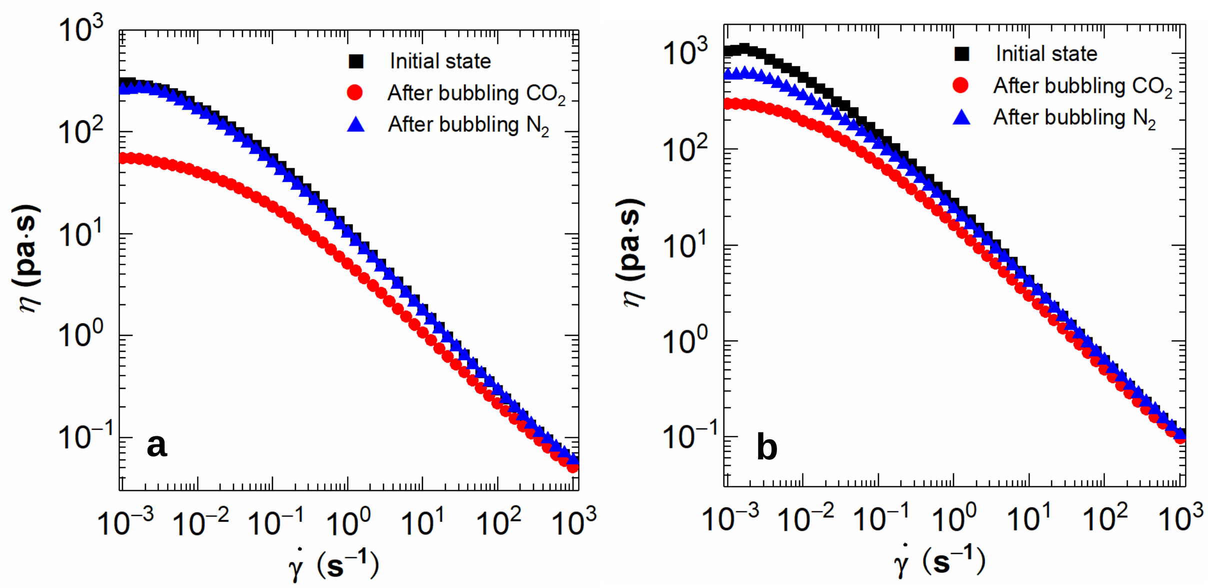

Figure S2 Viscosity-shear rate curves of the initial (a) 0.65 wt% and (b) 1.30 wt% NaPAA solution, and after bubbling CO_2 and then N_2 at 25 ºC.

S3: Strain sweep measurement of 0.65 wt% and 1.30 wt% NaPAA aqueous solutions

To confirm the linear viscoelastic region of 0.65 and 1.30 wt% NaPAA aqueous solution at 25 ºC, we conducted the stress sweep test of the two samples (Figure S3). Generally, the ultimate value of viscoelastic region, i.e. linearity limit (γ_L), is defined as the stress value when *G*' deviates from its stable value by 5% (ISO 6721-10 and ED/DIN EN 14770). It can be found that the γ_L of 0.65 wt% and 1.30 wt% NaPAA aqueous solution is all situated at 40 %. In other words, only when $\gamma < 40\%$, can the fluids keep a stable structure.

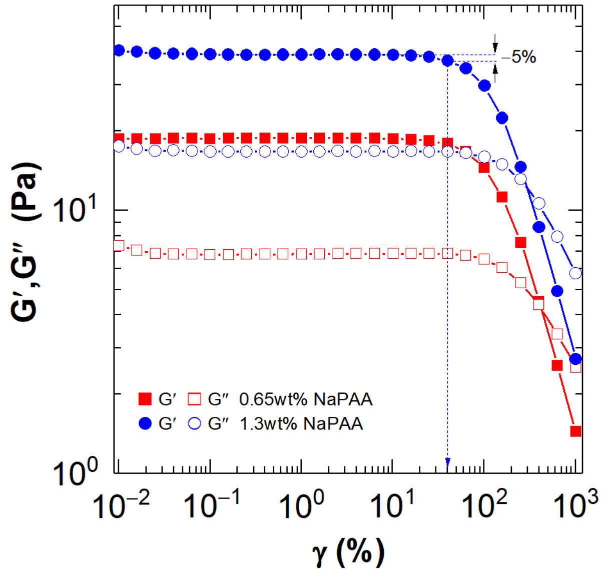

Figure S3 Strain sweep curves of 0.65 wt% and 1.30 wt% NaPAA aqueous solution at 25 ºC, ω = 10 rad·s^{-1}.

S4: Viscosity-shear rate curves of 0.65 wt% NaPAA solution with DMEA at different conditions

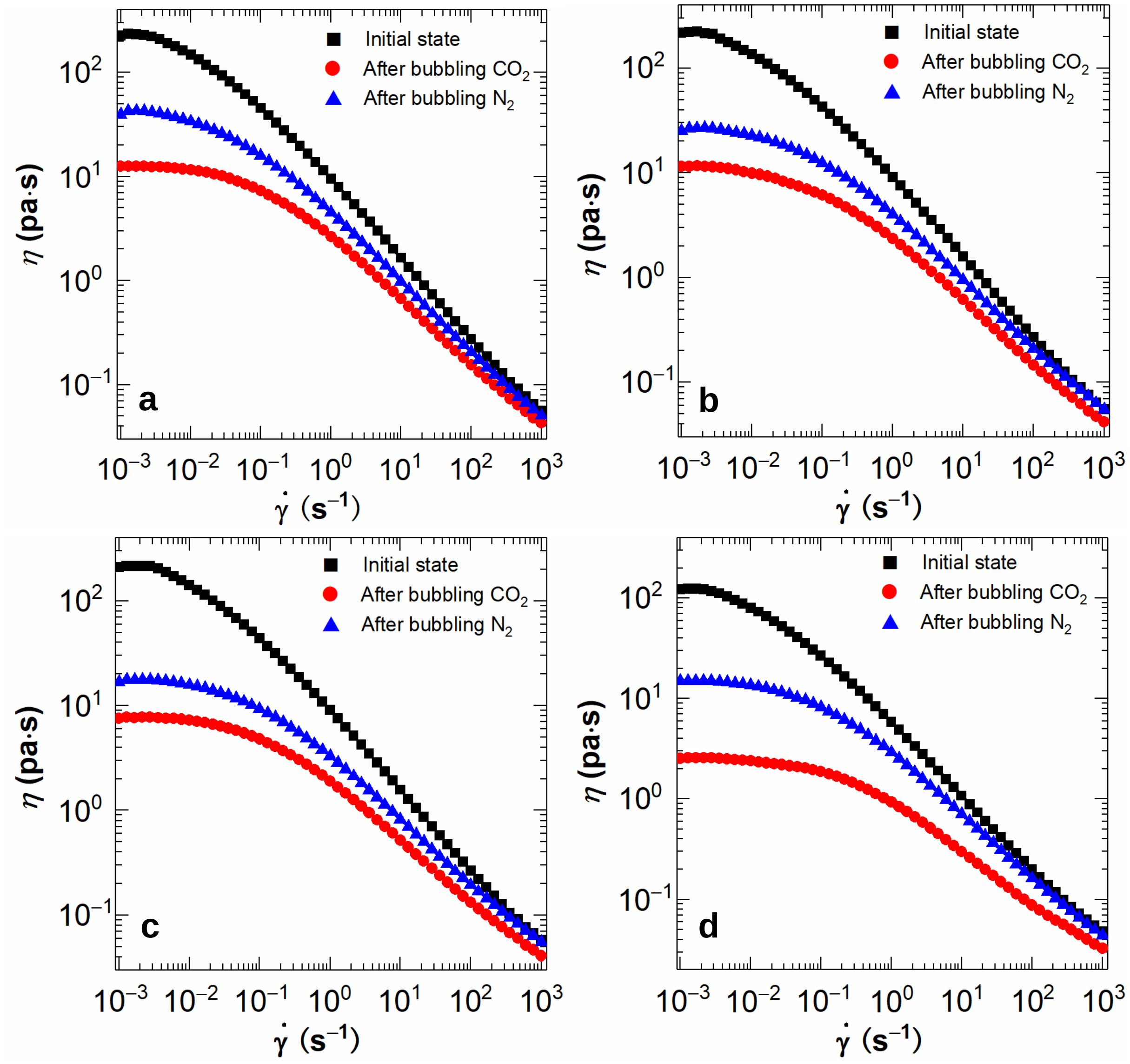

Figure S4 Viscosity-shear rate curves of 0.65 wt% NaPAA solution with (a) 0.6 wt%, (b) 1.2 wt%, (c) 2.4 wt% and (d) 4.8 wt% DMEA at 25 ºC.

S5: Viscosity-shear rate curves of 1.30 wt% NaPAA solution with DMEA at different conditions

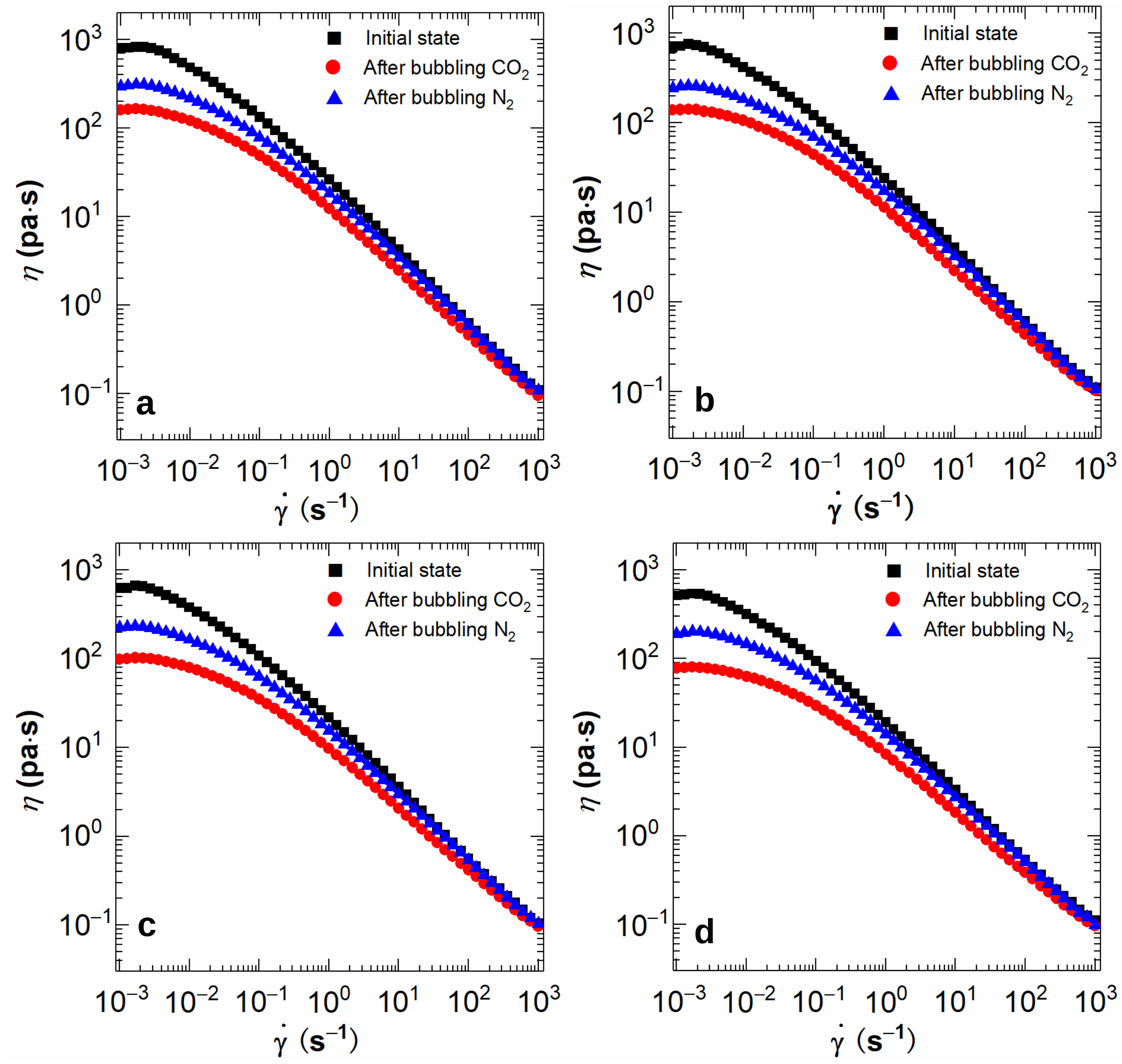

Figure S5 Viscosity-shear rate curves of 1.30 wt% NaPAA solution with (a) 1.2 wt%, (b) 2.4 wt%, (c) 4.8 wt% and (d) 9.6 wt% DMEA at 25 ºC.

S6: Loss and recovery ratio of η_0

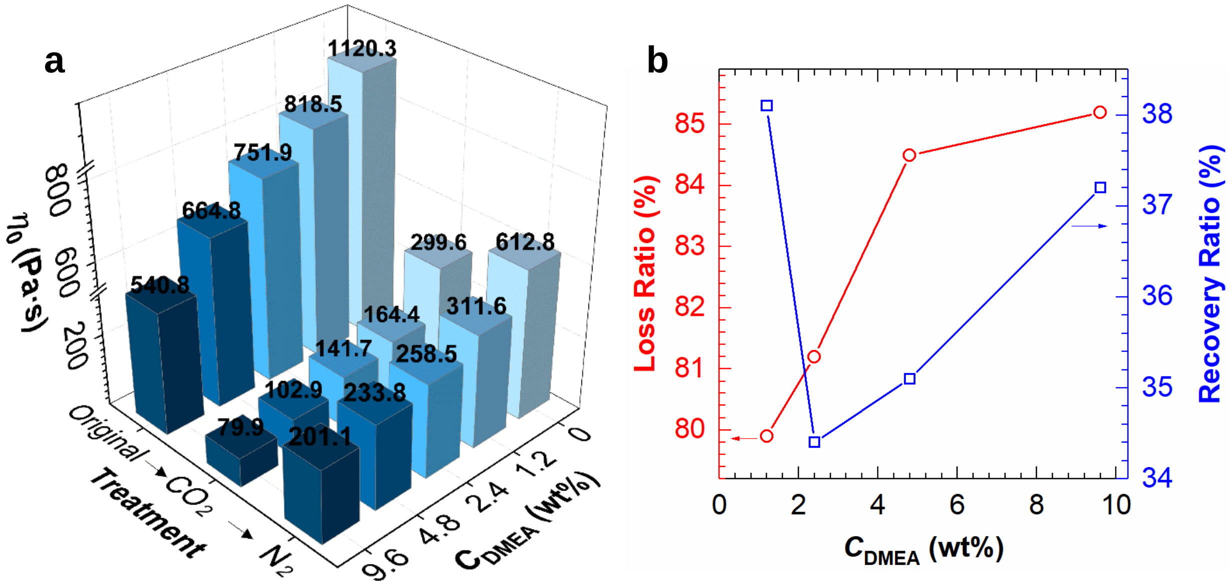

Figure S6. (a) η_0 of the initial 1.30 wt% NaPAA solution with different DMEA concentration, and after bubbling CO_2 and then N_2 at 25 ºC. (b) The loss ratio of η_0 of the solution after CO_2 treatment and the recovery ratio of η_0 of the solution after N_2 treatment.

As shown in **Figure S6**a, the initial η_0 of the 1.30 wt% NaPAA aqueous solutions was 1120.3 Pa·s^{-1}, then it dropped to 299.6 Pa·s^{-1} when bubbling CO_2; after introducing N_2 to remove CO_2, the η_0 recovered to 612.8 Pa·s^{-1}. Adding DMEA into the 1.30 wt% NaPAA aqueous solutions, the initial η_0 of the mixture decreased from 818.5 Pa·s^{-1} to 540.8 Pa·s^{-1} with the concentration of DMEA increased from 1.2 wt% to 9.6 wt%. In the presence of CO_2, the ammonium bicarbonate salts, *i.e.*, DMEAH$^+$, largely reduced the η_0 of the mixture. Specifically, its η_0 decreased from 164.4 Pa·s^{-1} to 79.9 Pa·s^{-1} (Figure S6a), and its loss ratio increased from 79.9 % to 85.2 % (Figure S6b). It can be found that the reduced degree in loss ratio of 1.30 wt% NaPAA/DMEA solution is lower than that of 0.65 wt% NaPAA/DMEA solution, which may be interpreted as the van der Waals force between molecular chins in 1.30 wt% NaPAA solution is stronger than that of 0.65 wt% solution, which can partially offset the viscosity loss induced by protonated DMEA. After treated with N_2, the η_0 of 1.30 wt% NaPAA/DMEA solution again recovered to close to its initial value (Figure S6a), and its loss ratio varied from 34.4 % to 38.1 % (Figure S6b).

S7: Frequency sweep curves of 1.30 wt% NaPAA aqueous solution with different C_{DMEA}

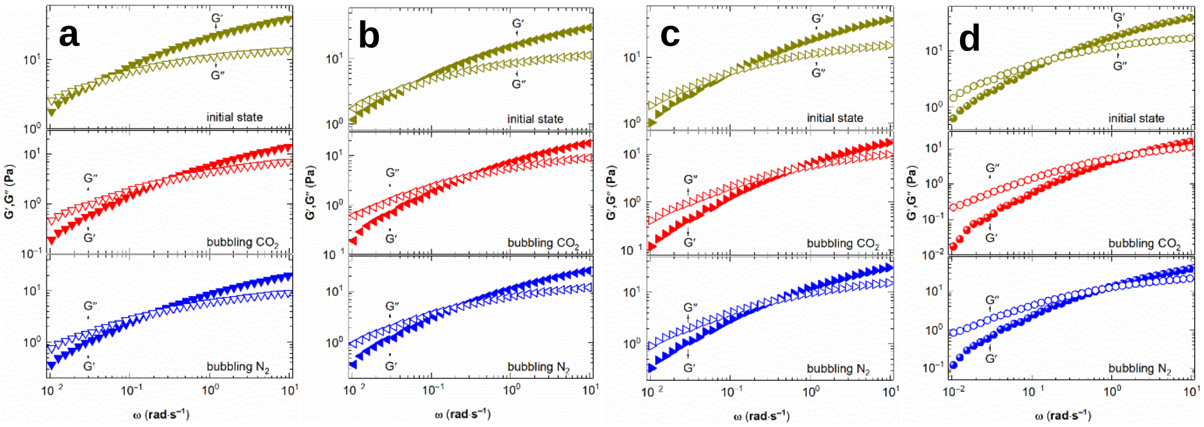

Figure S7. Frequency sweep curves of 1.30 wt% NaPAA aqueous solution containing (a) 1.2 wt%, (b) 2.4 wt%, (c) 4.8 wt%, and (d) 9.6 wt% DMEA in the presence or absence of CO_2, γ = 10%, T = 25 ºC.

S8: pH variation of 0.65 wt% and 1.30 wt% NaPAA aqueous solutions with different DMEA concentrations

Table S1 pH of 0.65 wt% NaPAA solution with different DMEA concentrations at different conditions

C_{DMEA} (wt%)	Initial	Bubbling CO_2	Bubbling N_2
0	9.17 ± 0.02	5.82 ± 0.03	7.99 ± 0.02
0.6	11.14 ± 0.03	6.75 ± 0.04	8.94 ± 0.02
1.2	11.26 ± 0.01	6.95 ± 0.03	8.93 ± 0.04
2.4	11.43 ± 0.02	7.25 ± 0.03	8.94 ± 0.04
4.8	11.57 ± 0.05	8.08 ± 0.02	9.21 ± 0.02

Table S2 pH of 1.30 wt% NaPAA solution with different DMEA concentrations at different conditions

C_{DMEA} (wt%)	Initial	Bubbling CO_2	Bubbling N_2
0	9.47 ± 0.04	6.31 ± 0.05	7.47 ± 0.12
1.2	11.36 ± 0.01	6.83 ± 0.04	8.18 ± 0.04
2.4	11.62 ± 0.05	7.14 ± 0.04	8.92 ± 0.02
4.8	11.83 ± 0.03	7.99 ± 0.01	9.17 ± 0.05
9.6	11.88 ± 0.02	8.14 ± 0.03	9.38 ± 0.02

S9: Conductivity variation of 0.65 wt% and 1.30 wt% NaPAA aqueous solutions with different DMEA concentrations

Table S3 Conductivity of 0.65 wt% NaPAA solution with different DMEA concentrations at different conditions

C_{DMEA} (wt%)	Initial	Bubbling CO_2	Bubbling N_2
0	2.06 ± 0.03	3.53 ± 0.01	2.54 ± 0.02
0.6	2.21 ± 0.02	7.39 ± 0.01	5.12 ± 0.01
1.2	2.36 ± 0.04	10.88 ± 0.02	6.42 ± 0.10
2.4	2.44 ± 0.03	17.31 ± 0.01	9.54 ± 0.05
4.8	2.47 ± 0.05	23.46 ± 0.12	12.86 ± 0.11

Table S4 Conductivity of 1.30 wt% NaPAA solution with different DMEA concentrations at different conditions

C_{DMEA} (wt%)	Initial	Bubbling CO_2	Bubbling N_2
0	3.86 ± 0.04	5.56 ± 0.02	4.32 ± 0.12
1.2	3.92 ± 0.03	11.24 ± 0.01	7.33 ± 0.04
2.4	4.05 ± 0.06	15.17 ± 0.02	8.77 ± 0.07
4.8	4.23 ± 0.05	22.33 ± 0.03	13.76 ± 0.21
9.6	4.92 ± 0.07	31.28 ± 0.16	15.56 ± 0.13

References

1. Castillo-Tejas J, Castrejón-González O, Carro S, González-Coronel V, Alvarado J, Manero O. Associative polymers. part III: shear rheology from molecular dynamics. *Colloid Surface., A*. **2016**, *491*, 37–49.
2. Zhu R, Feng Y, Luo P. Net Contribution of hydrophobic association to the thickening power of hydrophobically modified polyelectrolytes prepared by micellar polymerization. *Macromolecules*. **2020,** *53*, 1326–1337.
